# Cancer metabolism and carcinogenesis

**DOI:** 10.1186/s40164-024-00482-x

**Published:** 2024-01-29

**Authors:** Jianqiang Yang, Chloe Shay, Nabil F. Saba, Yong Teng

**Affiliations:** 1 Department of Hematology and Medical Oncology, Winship Cancer Institute, Emory University School of Medicine, 201 Dowman Dr, Atlanta, GA 30322 USA; 2 Wallace H. Coulter Department of Biomedical Engineering, Georgia Institute of Technology and Emory University, Atlanta, GA 30322 USA

**Keywords:** Metabolic heterogeneity, Metabolic exchange and integration, Metabolic patterns and regulation, Tumor heterogeneity, Heterogeneous treatment effect

## Abstract

Metabolic reprogramming is an emerging hallmark of cancer cells, enabling them to meet increased nutrient and energy demands while withstanding the challenging microenvironment. Cancer cells can switch their metabolic pathways, allowing them to adapt to different microenvironments and therapeutic interventions. This refers to metabolic heterogeneity, in which different cell populations use different metabolic pathways to sustain their survival and proliferation and impact their response to conventional cancer therapies. Thus, targeting cancer metabolic heterogeneity represents an innovative therapeutic avenue with the potential to overcome treatment resistance and improve therapeutic outcomes. This review discusses the metabolic patterns of different cancer cell populations and developmental stages, summarizes the molecular mechanisms involved in the intricate interactions within cancer metabolism, and highlights the clinical potential of targeting metabolic vulnerabilities as a promising therapeutic regimen. We aim to unravel the complex of metabolic characteristics and develop personalized treatment approaches to address distinct metabolic traits, ultimately enhancing patient outcomes.

## Introduction

Cancer metabolism was first established when Otto Warburg observed cancer cells using aerobic glycolysis instead of oxidative phosphorylation (OXPHOS) despite the presence of oxygen [1, 2]. This rewiring of metabolism enables the continuous growth and division of cancer cells and ensures an adequate supply of building blocks for cellular components. Since then, cancer metabolism has expanded to cover topics like glucose, fatty acid, nucleotide, and amino acid synthesis. It has been shown that the cellular reprogramming of cancer cells is due to the upregulation of oncogenes and downregulation of tumor suppressor genes. Cancer cells exploit this metabolic reprogramming to produce macromolecules and oncometabolites [3]. This leads to the six hallmarks of cancer, such as deregulated consumption of glucose and amino acids, alterations of metabolic gene-driven regulations, and glycolysis/ tricarboxylic acid (TCA) cycle intermediates being used for macromolecule synthesis [4, 5]. Cancer cells need to undergo metabolic reprogramming to efficiently adapt their cellular bioenergetics to the unfavorable conditions in tumor microenvironments (TME), characterized by low oxygen, high oxidative stress, acidity, and limited nutrients. Metabolic adaptation is widely acknowledged as a distinguishing characteristic of cancer cells. The study of cancer metabolism has evolved into a vibrant area of research, encompassing a wide range of innovative approaches to target metabolic pathways in cancer. Nonetheless, most studies do not consider the cellular diversity found within tumors. It is crucial to emphasize that various phenotypes, such as those associated with low oxygen levels versus normal oxygen levels or dormant versus actively dividing cells, will have distinct metabolic needs. Consequently, responses to metabolic therapies may vary significantly depending on these disparate requirements [6]. In this review, we will discuss cancer cell metabolic heterogeneity and the different cells in the cancer cell family and summarize the current research status of drugs targeting tumor metabolism.

## Metabolic reprogramming and transition

Metabolic reprogramming is considered a hallmark of tumorigenesis and progression and is influenced by a variety of factors. It involves changes in the utilization of different nutrients, specific demands of the cell, tissue of origin, potential carcinogenic and epigenetic changes, and cell-cell/cell-matrix interactions within the TME [7]. Metabolic reprogramming can also be considered one of the key factors in tumor development [8], based on transformation of the metabolic mode that causes cancer cells’ different physiological habits and survival modes [9].

**Fig. 1:**
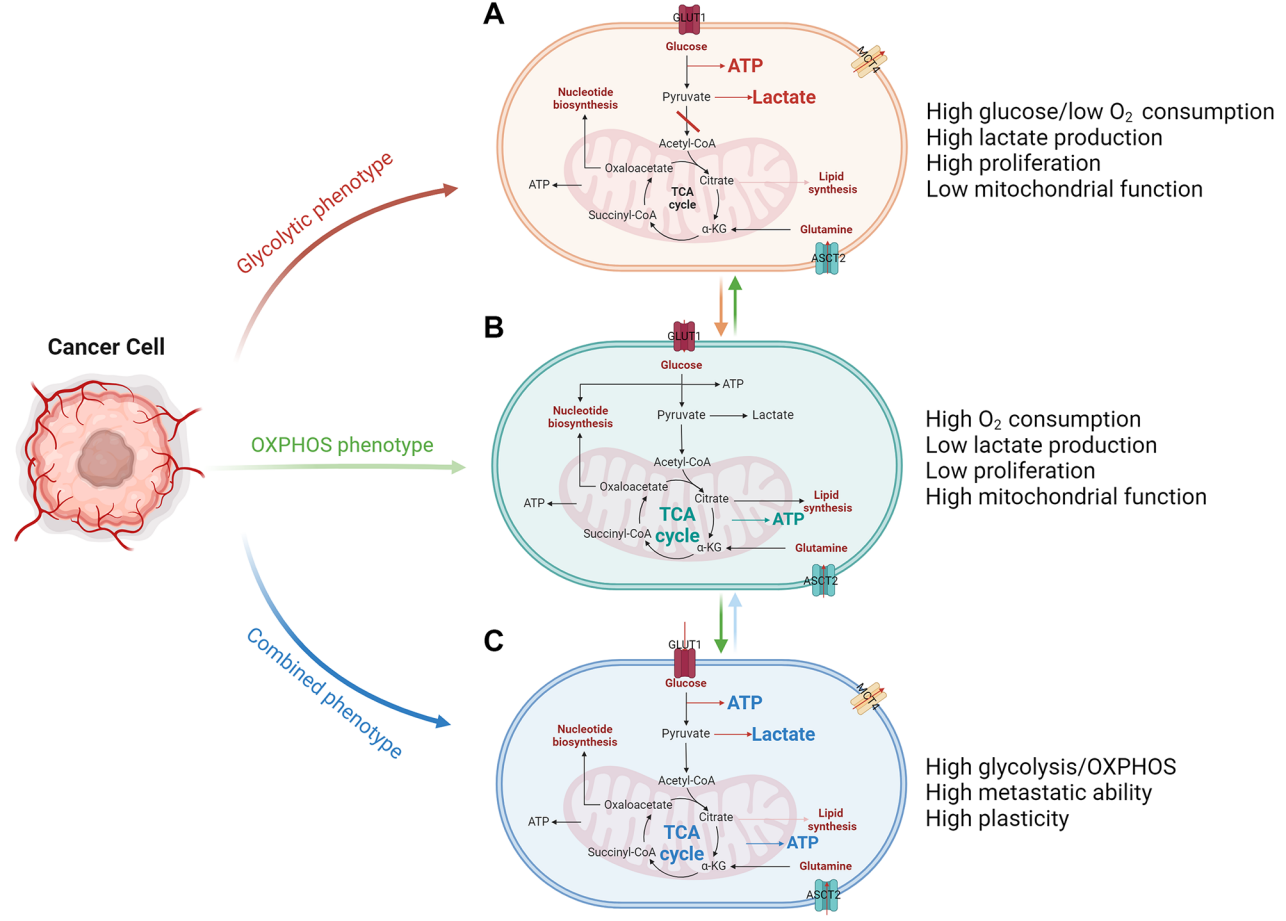
Metabolic patterns and their continuous transition in cancer cells. These metabolic patterns can transition from one to another according to different triggers and requirements in cancer cells. (**A**) Schematic representation of glycolytic metabolic phenotype. Cancer cells in the glycolytic metabolic pattern take up high levels of glucose and produce high levels of lactate to meet energy and synthesis demands. (**B**) Schematic representation of OXPHOS metabolic phenotype. Cancer cells have a better “burn efficiency” by relying on high mitochondrial function and consuming more oxygen. (**C**) Schematic representation of combined metabolic phenotype. Cancer cells in the metabolic pattern have high plasticity and characteristics of high glycolysis and mitochondrial metabolism

The carbohydrate metabolism of cancer cells includes three main types: aerobic glycolysis, oxidative phosphorylation, and a hybrid mode (Fig. 1). It is worth noting that these three modes are not constant and can undergo changes depending on the specific state of the cells and external stimuli [10]. Similarly, lipid and amino acid metabolism also can be altered [11]. Aerobic glycolysis is the most common mode for most cancer cells because it provides just the right amount of energy and many synthetic substances to support rapid proliferation [12]. Aerobic glycolysis allows cancer cells to run lightly without consuming too much oxygen and is also conducive to tumor growth and metastasis. Cancer cells take up glucose from the TME and produce lactate, which is excreted outside the cells [13, 14]. In glycolysis, some important glycolytic intermediates are altered. For example, cancer cells exhibit aberrant glycosylation, indicating alterations in the hexosamine biosynthetic pathway (HBP). HBP has been shown to influence many aspects of tumor biology, including metastasis development [15]. In addition, increased flux through the pentose phosphate pathway (PPP) produces more NAPDH to meet bioenergetic demands [16].

Most cancer cells rely on the aerobic glycolysis pathway for their energy source, while some rely on OXPHOS [17]. It is commonly believed that when the metabolic rate of cancer cells is equal to or lower than that of normal tissues, such as in cancer stem cells (CSCs), cancer cells transition to the OXPHOS pathway [18, 19]. The advantage of this pathway is that it can provide more energy for cancer cells that do not require extensive proliferation, allowing a more efficient “burning” of nutrients. This mechanism can help cells maintain quiescence without competing for nutrients [20]. Similarly, some cancer cells are in a hybrid state, which is often considered a transitional state between the two pathways. It is generally believed that CSCs undergo this hybrid state during the transition [21]. Proliferating CSCs rely on both glycolysis and OXPHOS. Furthermore, through the reverse Warburg effect, quiescent and proliferating CSCs can use catabolites from cancer-associated stromal cells [22].

### The metabolic characteristics of nutrients in cancer cells

#### Glycolysis and TCA cycle alterations

Cancer cells have altered metabolism compared to normal cells, and one key difference is the upregulation of glycolysis and alterations in the TCA cycle. Because of the Warburg effect in cancer cells, glycolysis is upregulated and generates ATP and biosynthetic precursors, as cells have increased energy demands for rapid proliferation. The pyruvate produced by glycolysis is largely converted into lactate instead of entering the TCA cycle, even in the presence of oxygen [23]. This lactate production helps balance reducing equivalents and enables cancer cells to continue glycolysis. While the Warburg effect continues, the lactate produced is not a useless waste product. Lactate is not only important for tumor invasion, metastasis, and angiogenesis [24, 25], but also has immunosuppressive functions, such as inducing and recruiting immunosuppressive cells and molecules, thereby promoting tumor development and escape [26, 27]. Cancer cell-derived lactate can induce the expression of vascular endothelial growth factor (VEGF) and arginase 1 (Arg1) through HIF-1α signaling pathway, which promotes the polarization of tumor-associated macrophages (TAMs) toward the M2 phenotype, enabling TAMs to promote tumor growth [28]. In addition, some studies have shown that lactate can increase the potency of CD8^+^ T cells, thereby enhancing anti-tumor immune responses [29, 30]. Notably, mitochondrial metabolism also plays a critical role in fueling tumor growth by providing essential metabolites for the synthesis of large molecules and generating oncometabolites to sustain cancer phenotypes [31]. Alterations in the TCA cycle occur in cancer cells. Various enzymes and metabolites involved in the TCA cycle may be disrupted, leading to abnormal metabolism. One example is downregulation or loss of succinate dehydrogenase (SDH) in certain cancers [32]. SDH is a key enzyme in the TCA cycle that converts succinate to fumarate, and its loss disrupts the normal flux of metabolites through the cycle. Mutations in the genes encoding SDH subunits have been found in several types of cancer, including paragangliomas, pheochromocytomas, and gastrointestinal stromal tumors [33]. Another alteration in the TCA cycle in cancer cells is the accumulation of intermediate metabolites, such as citrate and fumarate [34]. This accumulation may result from genetic or epigenetic alterations in enzymes that regulate the TCA cycle, such as isocitrate dehydrogenase (IDH) or fumarate hydratase (FH). Fumarate may have direct effects on cell signaling pathways involved in cell growth and survival and has been shown to activate the mammalian target of the mTOR pathway [35]. Loss of FH activity leads to fumarate accumulation and affects cellular metabolism and energy production, potentially promoting cancer cell growth and survival. The alterations in glycolysis and the TCA cycle play a critical role in cancer cell metabolism, enabling cancer cells to adapt to their increased energy demands and support rapid proliferation. Targeting these metabolic alterations has emerged as a promising strategy for cancer therapy, with several drugs currently in development that selectively target metabolic pathways in cancer cells (Table 1) [36].

**Table 1:** Functional and representative inhibitors of metabolic targets

Targeting molecules	Function	Agents
GLS1	Metabolize glutamine into glutamate	CB-839 [37], LWG-301, IPN60090
ASCT2	Transport neutral amino acids	V9302 [38], SN40, SN02
LAT1	Transport neutral amino acids	BCH [39], KMH-233, GPNA hydrochloride
GLUT1	Transport glucose	BAY-876, WZB117, STF-31 [40]
GLUT4	Transport glucose, insulin dependent	Fasentin [41], GLUT4-IN-2
MCT1	Transport and import lactate	BAY-8002, AZD3965 [42]
MCT4	Transport and export lactate	VB124, Syrosingopine [43], AZD0095
PFK-2	Catalyze fructose-2,6-bisphosphate	PFK-015 [44]
FASN	Synthesize fatty acid	TVB-2640, UCM05, Trans-C75
ACC	Catalyze the carboxylation of acetyl-CoA to produce malonyl-CoA	TOFA [45], ND-646
CPT1	Catalyze the transport of long-chain fatty acids into mitochondria	Etomoxir [46]
Mitochondrial Complex I	The major entry point for electrons into the respiratory chain	IACS-010759 [47], DX2-201, HQNO, SCAL-266

#### Lipid metabolism alterations

Akin to glycolysis and TCA cycle alterations, lipid metabolism is another metabolic reprogramming target in cancer. It has been shown that de novo fatty acid synthesis is a hallmark of cancer. These fatty acids serve as important macromolecules for membrane stability, energy supply, and as a cell signaling molecule. Regarding membrane stability, it is important to note that membrane saturation and rigidity play a role in chemotherapy and drug resistance. Higher membrane saturation gives way to less oxidative stress induced by chemotherapy, and increased cholesterol increases membrane rigidity, thus decreasing its permeability to drug treatment [48]. Cancer cells increase lipogenesis and require more lipid modification to survive. One important modification is lipid desaturation, an important process of adding double bonds to the acyl chain of the fatty acid, which plays a critical role in the biosynthesis of lipids. Lipid desaturation increases the fluidity and flexibility of the cell membrane, facilitating membrane remodeling and the trafficking of membrane-associated proteins and lipids, thereby promoting plasticity, migration, invasion and survival [49–51]. An important desaturase in the process of lipid desaturation is stearoyl-CoA desaturase 1 (SCD1). SCD1 promotes lipid mobilization in subcutaneous white adipose tissue. SCD1 plays a crucial role in transforming saturated fatty acids (SFAs) into monounsaturated fatty acids (MUFAs), serving a key function in preserving membrane fluidity, cellular signaling, and gene expression. Elevated expression of SCD1 has been observed in numerous malignancies, and higher levels of SCD1 are associated with more aggressive tumor behavior, poorer prognosis and increased resistance to chemotherapy [52]. Additionally, there are key enzymes in fatty acid synthesis. One such important enzyme is fatty acid synthase (FASN), the rate-limiting enzyme in synthesizing fatty acids, which can be induced via AKT and HIF-1 under hypoxic stress, allowing for adaptations in the TME [53]. Targeting FASN also represents a new therapeutic opportunity for patients with breast cancer and brain metastases [54]. There is conflicting evidence regarding the purpose of fatty acid reprogramming, with some pointing to increased endogenous fatty acid synthesis as a pathway for cancer progression and tumorigenesis via membrane biogenesis [55, 56], while others concluding the opposite [57]. A study by Hopperton et al. showed that breast cancer cells do not rely solely on endogenous synthesis of fatty acids because exogenous palmitate is also predominately incorporated into phospholipids [58].

#### Amino acid metabolism alterations

**Fig. 2:**
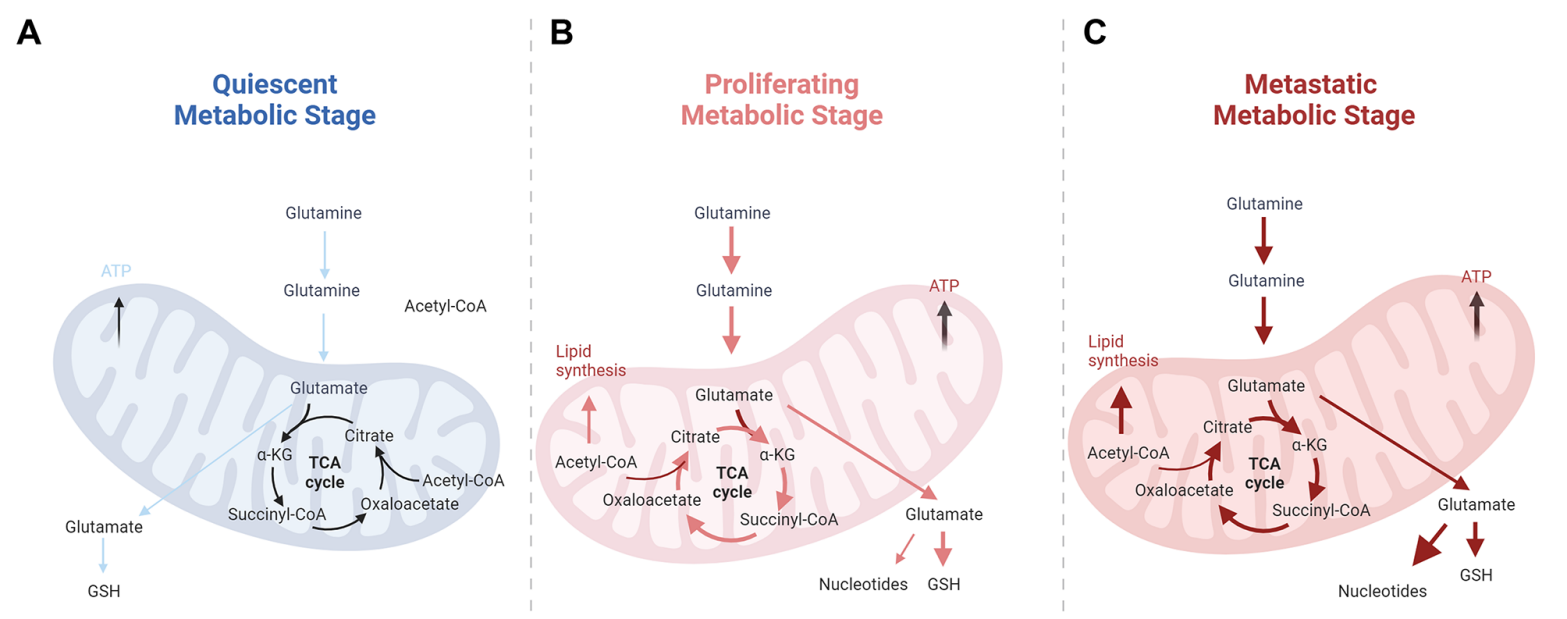
Glutaminolysis at different stages of the metabolism. Glutamate is converted to the TCA cycle intermediate α-KG and the corresponding amino acid. The newly formed citrate exits the mitochondria where it is used to synthesize fatty acids and amino acids, transfer glutamate to the cytoplasm, and synthesize GSH, which is critical for maintaining redox homeostasis and protecting cells from oxidative stress. a broad upregulation of biosynthetic pathways characterizes proliferative and metastatic metabolism by glutamine. (**A**) In quiescent metabolism, glutamine metabolism is maintained at a low level. (**B**) In the proliferative stage, glutamine consumption has increased and more GSH is needed to counteract oxidative stress. (**C**) When cancer cells transform to the metastatic stage, more lipids and nucleotides are synthesized to adapt to the synthetic needs

Glutamine is an essential amino acid that plays a crucial role in cancer cells as they have a higher demand for glutamine than normal cells due to their rapid growth and proliferation (Fig. 2) [59]. Glutamine can be used as a building block for synthesizing proteins, nucleotides, and other macromolecules necessary for cell division. Glutamine can also be used as a substrate in the TCA cycle, a key metabolic pathway that generates energy in the form of ATP. Moreover, glutamine metabolism in cancer cells can contribute to the replenishment of the cellular antioxidant defense system. Cancer cells often face oxidative stress due to rapid proliferation and elevated metabolic activities. Glutamine can produce glutathione, an important antioxidant that protects cells from oxidative damage. Cancer cells can obtain glutamine without nutrients by breaking down large molecules [60]. For example, overactivation of the oncogene RAS promotes endocytosis, in which cancer cells clear extracellular proteins and degrade them into amino acids such as glutamine, providing nutrients to cancer cells [61]. Targeting glutamine metabolism may become a new cancer treatment approach. It has been found that blocking glutamine can induce different metabolic processes to overcome tumor immune escape [62].

Glutamine is critical for cancer cells as it is a major carbon and nitrogen source for biosynthetic processes. Cancer cells often display increased uptake and utilization of glutamine to support cell growth and proliferation. Glutamine can be converted to glutamate and metabolized to α-ketoglutarate, an intermediate in the TCA cycle. In addition, cancer cells take up many other amino acids to support their rapid proliferation [63]. Serine and glycine are two amino acids that are critical for cells and support nucleotide synthesis, protein synthesis, and redox balance [64, 65]. Tryptophan is another essential amino acid that can be metabolized through different pathways, including the kynurenine pathway [66]. In cancer cells, tryptophan metabolism may be altered, leading to increased production of metabolites involved in immunosuppression and immune evasion. Cancer cells can increase the uptake of branched-chain amino acids (BCAAs), including leucine, isoleucine, and valine, to support their rapid proliferation. Leucine, in particular, can activate the mammalian target of rapamycin (mTOR) pathway, which is involved in cell growth and protein synthesis [67]. In particular, the urea cycle (UC) is thought to remove toxic ammonia from the body by converting it to urea, which can be excreted in the urine. High levels of ammonia lead to neurotoxicity, yet cancer cells recycle ammonia and reuse it for amino and nucleic acid synthesis [68]. Alterations in amino acid metabolism in cancer cells contribute to their metabolic rewiring and provide the necessary building blocks and energy for their high proliferative and invasive behavior. These metabolic alterations can be targeted for therapeutic interventions, such as using inhibitors targeting glutamine metabolism or amino acid transporters to kill cancer cells or inhibit their growth selectively [69, 70].

## The metabolic characteristics of CSCs

Growing evidence supports the theory of CSCs as an important mechanism by which existing therapies fail to eradicate cancer [71]. In addition to having a more defined role in maintaining minimal residual lesions and forming new tumor bodies after treatment, CSCs may also play a key role in tumor recurrence and metastasis. Therefore, specific clearance of CSCs may be among the most important therapeutic strategies. Emerging evidence suggests CSCs have a different metabolic phenotype from differentiated cancer cells [72]. These specific metabolic activities directly involve CSCs’ transformation or support biological processes that enable tumor progression.

As the core of cancer, CSCs show intricate metabolism. Several researchers have suggested that CSCs are more glycolytic than other differentiated cancer cells in vitro and in vivo, and are found in many tumor types, including osteosarcoma, breast, lung, and colon cancers, and others [73–75]. Compared with differentiated cancer cells, glucose uptake, glycolytic enzyme expression, lactic acid production, and ATP were significantly increased in CSCs [76]. This glycolytic phenotype appears to be associated with reduced mitochondrial oxidative metabolism.

However, emerging evidence indicates that CSCs preferentially rely on mitochondrial oxidative metabolism [77, 78]. These studies suggest that CSCs exhibit lower glycolytic activity, consume less glucose, produce less lactate, and maintain higher ATP levels than their differentiated progeny. Additionally, the mitochondria of CSCs have increased mass and membrane potential, indicating enhanced mitochondrial function. CSCs also demonstrate higher mitochondrial reactive oxygen species (ROS) levels and oxygen consumption rates than differentiated cancer cells [79–81]. The increased mitochondrial mass in CSCs is associated with stem-like traits, metastatic potential, and resistance to DNA damage. Invasive migratory cancer cells also exhibit high mitochondrial metabolism by activating PGC1α, a mediator of mitochondrial biogenesis [82]. PGC1α is overexpressed in circulating cancer cells and is necessary for OXPHOS in a subset of human melanomas. Inhibition of PGC1α reduces the stemness properties of breast CSCs [72]. Similarly, pancreatic cancer cells with CSC features rely more on mitochondrial function and less on glucose and glutamine for survival [83]. Ovarian cancer CSCs have been found to overexpress genes associated with mitochondrial OXPHOS and fatty acid oxidation (FAO). This oxidative phenotype is related to CSC resistance to apoptosis [84]. Despite high levels of mitochondrial ROS in CSCs, total levels of ROS are significantly lower in CSCs compared to non-CSCs, and CSCs have a more robust antioxidant defense system [85]. This antioxidant response helps maintain the stemness and tumorigenic capacities of CSCs and contributes to therapy resistance [86]. Considering the mitochondrial metabolic characteristics, CSCs may upregulate NADPH production, which plays importance in maintaining the redox balance within cells by providing reducing power to counteract the harmful effects of ROS. Changes in lipid metabolism not only meet the energy and biomass needs of CSCs, but also play a role in the activation of key pro-cancer signaling pathways, such as the Wnt/β-catenin and Hippo/YAP pathways [87]. Lipids are an important source of energy for cells and are involved in various cellular processes, including membrane maintenance and signaling. CSCs have been shown to have a distinct lipid profile compared to non-CSCs, including increased levels of fatty acids, cholesterol and lipid droplets. Alterations in lipid and cholesterol-associated pathways are essential for the maintenance of CSCs [88]. High levels of lipid droplets have been reported in colorectal CSCs and breast CSCs [89, 90]. Furthermore, it was found that inhibition of fatty acid β-oxidation preferentially eliminates the CSC population, and that fatty acid β-oxidation is critical for self-renewal and chemoresistance of breast CSCs [91]. Additionally, CSCs exhibit elevated amino acid metabolism to meet the demands of rapid cell proliferation and cellular homeostasis According to the findings of Jones and colleagues, leukemia stem cells have increased amino acid metabolism, and inhibiting amino acid uptake with specific inhibitors can lead to toxicity in leukemia stem cells [92].

### The metabolic characteristics of invasive leader cells

Invasive leader cells, also known as invasive front cells, are a subset of cancer cells critical in driving tumor invasion and metastasis [93, 94]. These cells are characterized by their ability to break away from the primary tumor mass, invade surrounding tissues, and initiate the formation of secondary tumors at distant sites. However, invasive leader cells are not constant and can change in response to external cues, which include the extracellular matrix (ECM), soluble factors, and neighboring cells. Invasive leader cells possess unique molecular and cellular features enabling invasive behavior. These cells often exhibit a more mesenchymal phenotype, undergo epithelial-mesenchymal transition (EMT), and have enhanced motility and the ability to degrade the ECM [95]. They secrete enzymes such as matrix metalloproteinases (MMPs) that can break down ECM components, facilitating their movement through tissues. Additionally, they can form invadopodia, specialized actin-rich protrusions that aid in ECM degradation and invasion, so they acquire enhanced migratory and invasive capabilities. Invasive leader cells rely on complex signaling networks to drive their invasive behavior [96]. They receive signals from the tumor microenvironment, including cues from cancer-associated fibroblasts (CAFs), immune cells, and the ECM. These signals can activate various signaling pathways, such as the Wnt, TGF-β, and PI3K-Akt pathways, which promote invasion and metastasis.

One key metabolic adaptation observed in invasive leader cells is enhanced mitochondrial respiration, which can enable the cells to meet the high energy demands associated with their invasive and migratory behavior [97, 98] through the following possible mechanisms: (1) Increased energy production: Invasion is a highly energy-demanding process that requires the cell to move and remodel its cytoskeleton to penetrate surrounding tissues actively. Mitochondrial respiration can offer a major source of ATP that drives cellular motility and invasive properties. (2) Metabolic flexibility: As the frontier of invasion, enhanced mitochondrial respiration is an adaption that can provide the cells with metabolic flexibility to respond to varying environmental conditions. (3) Regulation of redox balance: Mitochondrial respiration is tightly linked to the cellular redox balance by producing ROS as byproducts. Invasive leader cells may enhance mitochondrial respiration to regulate ROS production and maintain an optimal redox balance to support their invasive phenotype [99, 100]. The rearrangement of actin cytoskeletons contributes to invasion by forming protrusions such as invasive or pseudopods, and the formation of these structures depends on ROS signaling. Nox1-generated ROS drive signaling pathways, such as p38 MAPK and RHOA RhoA-associated protein kinase, which control the extent and direction of invasion [101].

It is generally believed that invading leader cells may reduce lipogenesis. Since invasion is an energetically demanding process that requires significant resources, reducing lipogenesis allows invading leader cells to allocate more energy towards other essential processes such as the formation of invadopodia and cytoskeletal remodeling. Lipogenesis requires the synthesis of fatty acids and their conversion into triglycerides for storage. This process involves various enzymes and transporters, which can impede the movement and flexibility of leader cells [102]. Decreasing lipogenesis helps redirect metabolic resources toward other pathways necessary for invasion, such as glycolysis and amino acid metabolism. Leader cells can enhance their motility and migrate more efficiently through the surrounding tissue. Based on this, targeting invasive leader cells is an active area of research for developing anti-metastatic therapies. By understanding the molecular and cellular mechanisms underlying their invasive behavior, researchers aim to identify specific targets or pathways that can be therapeutically targeted to suppress tumor invasion and metastasis [103, 104].

The heterogeneity within tumors and the presence of specialized cell populations such as CSCs and invasive leader cells underscore the complexity of cancer biology. While invasive leader cells share some metabolic similarities with CSCs, there are notable differences between the two. CSCs play a critical role in tumor initiation, growth and maintenance [105]. Leader cells, on the other hand, primarily display enhanced motility and the ability to interact with the ECM, and lack the extensive functions ascribed to CSCs [106]. CSCs have the unique ability to generate tumors from a minimal number of cells, an ability not shared by leader cells [107]. Leader cell formation is influenced by factors such as genetic heterogeneity, epigenetic states, and interactions within the tumor stroma. Leader cells are also closely associated with the collective invasion status and metabolic demands. In cases where the energy level of leader cells falls below a threshold, they are replaced by “follower cells” to maintain persistent invasion [108].

### The metabolic characteristics of trailing follower cells

Unlike leader cells, follower cells are in a relatively stable environment and communicate with leader cells through adhesion-based mechanical interactions; thus, follower cells support the leader cells to further expand the tracks. The primary pathway for energy production in follower cells is aerobic glycolysis, which is essential for their survival and function [109–111]. As mentioned above, high-throughput glycolysis can rapidly provide energy and raw materials for the synthesis of various biological macromolecules. In addition, in the process of aerobic glycolysis, a large amount of pyruvate is converted to lactic acid instead of entering the TCA cycle, reducing the production of ROS, which is beneficial in tumor development [112, 113]. Besides energy production, follower cells also engage in other metabolic processes to support their functions during invasion. Compared with leader cells, they must synthesize biomolecules such as lipids, nucleotides, and proteins to maintain cellular structure and function. For example, lipids are crucial components of cell membranes, and cancer cells need to generate new membranes as they divide and grow constantly [114]. Follower cells can acquire these building blocks by actively scavenging and utilizing nutrients in the tumor microenvironment. Follower cells also exhibit high metabolic plasticity to respond to changes in the environment.

In follower cells, lipogenesis is dysregulated and increased, leading to increased synthesis of fatty acids and lipids. This process is crucial as it provides the necessary building blocks for membrane synthesis, energy storage, and signaling molecules. Additionally, lipogenesis supports the high metabolic demands by providing a source of energy via FAO [115]. Lipogenesis is regulated by various factors, including oncogenes such as Myc, Akt, and AMPK, and tumor suppressor genes such as p53 [116, 117]. These alterations in gene expression and signaling pathways result in increased expression of lipogenic enzymes such as FASN, acetyl-CoA carboxylase (ACC), and ATP citrate lyase (ACLY), which drive lipogenesis in follower cells. Targeting lipogenesis in cancer cells has emerged as a potential therapeutic strategy [118]. Inhibition of lipogenic enzymes, such as FASN, has been shown to have anti-tumor effects in preclinical models of various cancers. Additionally, targeting lipogenesis in combination with standard chemotherapy or targeted therapies has shown synergistic effects and improved treatment outcomes [119].

### The metabolic characteristics of hypoxic core cancer cells

**Fig. 3:**
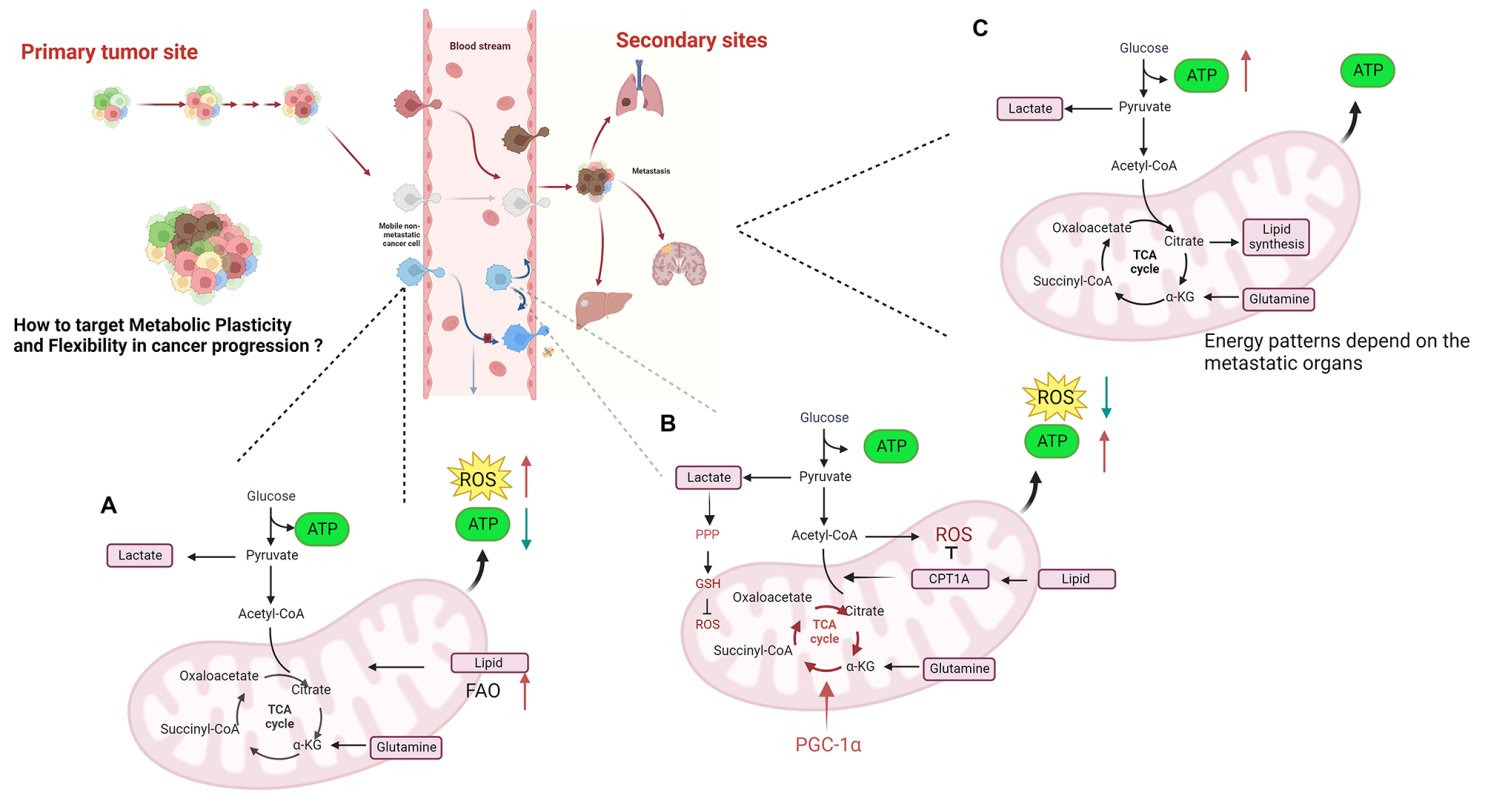
Metabolic switch during cancer cell invasion and metastasis. The metabolic pattern of cancer cells changes in response to various factors within the TME. (**A**) During preparation for invasion, there is an increase in ROS, a decrease in ATP synthesis, and activation of FAO to compensate for ATP synthesis. (**B**) During the metastatic process, increased TCA cycle and reduced ROS by CPT1A lead to increased ATP synthesis. (**C**) When cancer cells metastasize to target organs, there is a shift in the energy patterns that is influenced by the specific organ and its microenvironment

Due to rapid expansion of tumors, tumor cells situated approximately 150 μm away from the patient’s blood vessels may experience oxygen deprivation. Within the central region of the solid tumor, cells endure conditions characterized by insufficient oxygen and nutrients [120]. In this hypoxic core, cancer cell mitochondria experience impaired function, and as a result, glycolysis becomes the preferred energy generation pathway. These cells predominantly depend on “classical glycolysis” to produce energy. This pattern allows cancer cells in a hypoxic core to provide energy at a minimum for energy generation and survival under low-oxygen conditions. The switch to glycolysis in hypoxic core cancer cells has been associated with several other metabolic alterations, for example, glucose uptake from the extracellular environment and increased expression of glucose transporters on the cell surface [121]. Additionally, the expression of several glycolytic enzymes is upregulated, allowing for enhanced glucose metabolism. Importantly, due to long-term hypoxia, these cells still face the possibility of necrosis (Fig. 3).

### The challenges of studying metabolic plasticity

**Fig. 4:**
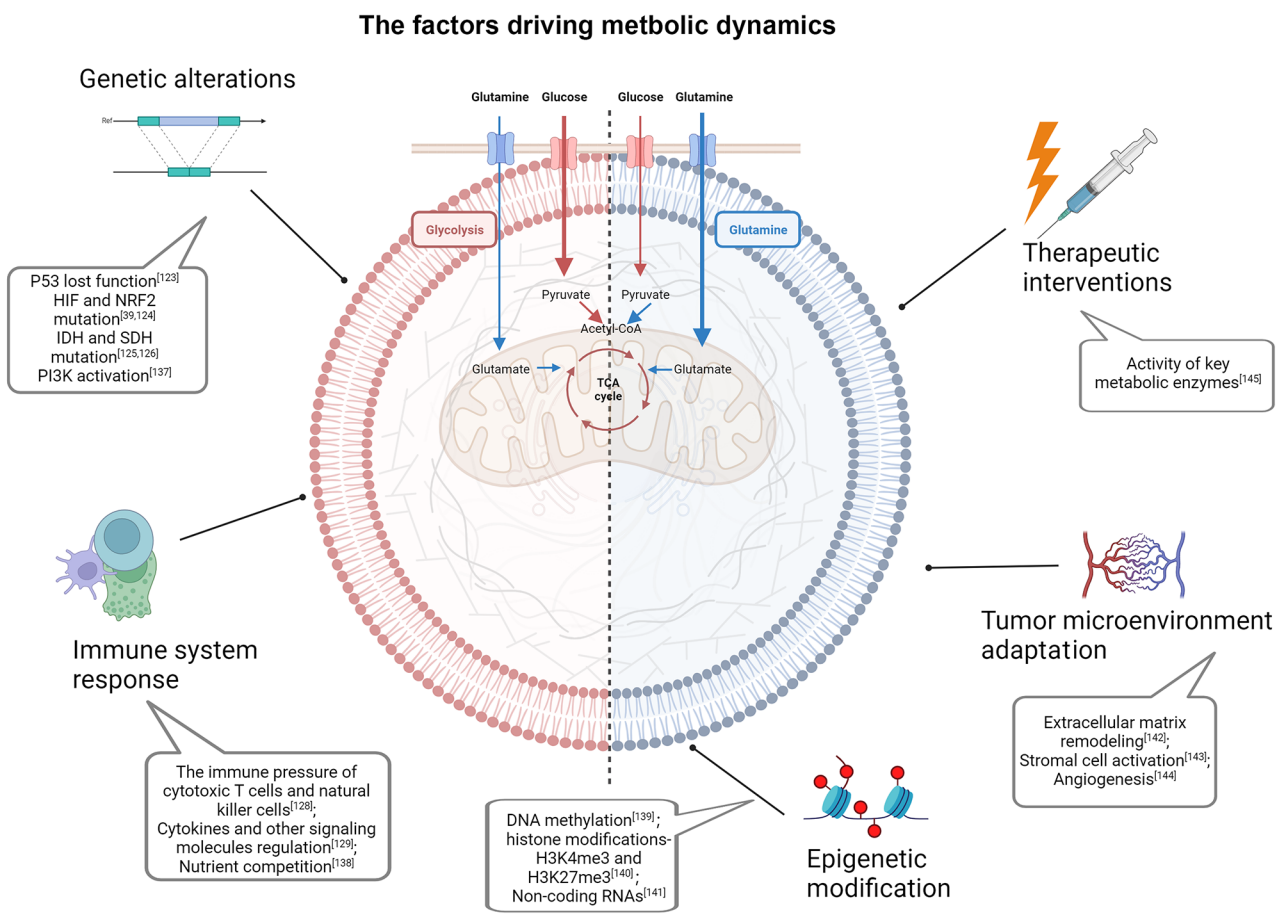
The factors driving metastatic dynamics. The metabolic dynamics of cancer cells are extremely complex and are influenced by multiple factors including genetic alterations, immune responses, epigenetic modifications, therapeutic interventions, and adaptations within the TME. The metabolic profiles of cancer cells are altered in response to single or multiple factors [39, 123–126, 128, 129, 137–145]

As mentioned above, metabolic plasticity is a hallmark of cancer cells in which they manipulate their metabolic profile to meet the dynamic energetic demands of the TME [122]. Although the understanding of metabolic plasticity has increased over the years, there are still many challenges to the study of metabolic plasticity. One of the challenges in the study of metabolic plasticity is the dynamics of metabolic plasticity. There are genetic alterations in cancer cells, especially some key gene mutations and oncogene activation [123–126], for example, transcription factors such as HIF and NRF2 can regulate the expression of enzymes causing metabolic changes [127]. Another important change is the therapeutic interventions, it can also induce metabolic enzymes abnormal activation. Metabolic networks can also adapt and rewire in response to changes in environmental conditions or cellular demands, including the immune pressure of cytotoxic T cells and natural killer (NK) cells [128] and the regulation of cytokines and other signaling molecules [129]. Thus, metabolic plasticity is not a static phenomenon, but a highly dynamic and responsive process. Understanding and characterizing these dynamic changes in metabolic networks is challenging. Another challenge is the complexity of metabolic networks and the interconnectedness of patterns. Increasing evidence supports the notion that epigenetic modification and TME adaptation contribute to the progression of metabolic changes (Fig. 4). Metabolites are constantly produced and consumed in a highly regulated manner, and changes in one part of the network can have ripple effects throughout the system, making it difficult to predict how a particular change in a metabolic enzyme or pathway will affect overall cellular metabolism. It has also been difficult to accurately measure and quantify changes in metabolite levels and fluxes using current techniques (Fig. 4).

### Targeting metabolic plasticity and flexibility dynamics for cancer therapy

**Fig. 5:**
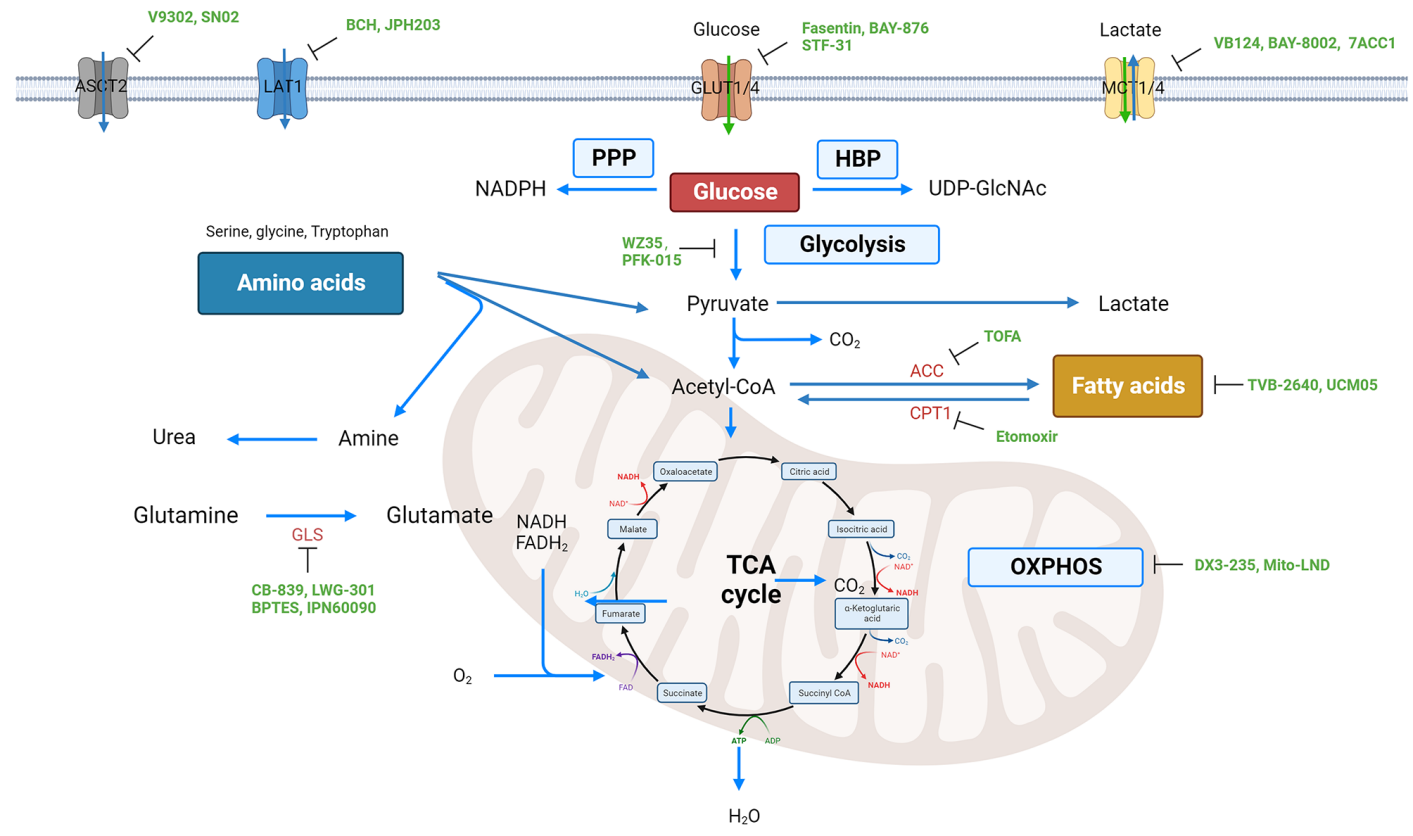
The main metabolic pathways for three key nutrients in cancer cells, along with their typical inhibitors. These inhibitors can be directed to impede the functioning of the pathways by targeting the relevant transporters and directly inhibiting the corresponding rate-limiting enzymes

Cancer therapies have made significant advancements in recent decades, especially with the rise of immunotherapy and targeted therapy [130, 131]. However, the emergence of drug resistance and toxicity remain major challenges in treatment. One emerging concept in cancer therapy is targeting metabolic plasticity and flexibility dynamics [132, 133]. As mentioned above, cancer cells exhibit enhanced metabolic flexibility, allowing them to switch between different nutrient sources and metabolic pathways depending on the availability of nutrients and the microenvironment (Fig. 4). Accordingly, targeting metabolic plasticity and flexibility dynamics in cancer therapy has emerged as a promising approach to overcome drug resistance and improve treatment efficacy. Understanding this concept can lead to the identification of several potential targets for therapy. One such target is the key metabolic enzyme hexokinase 2 (HK2), which plays a crucial role in glycolysis. Inhibiting HK2 can impair glycolytic flux, force cancer cells to reduce aerobic glycolysis, and rely on oxidative phosphorylation, which may increase their susceptibility to other therapies [134]. Another approach to target metabolic plasticity is inhibiting key signaling pathways that regulate nutrient uptake and metabolism. For example, the PI3K/Akt/mTOR pathway is often dysregulated in cancer and promotes metabolic reprogramming [135]. Targeting this pathway can disrupt cancer cell metabolism and sensitize cells to other therapeutic interventions. Certain inhibitors, like SN02 that hinders LAT1, BCH that impedes ASCT2, and Fasentin and BAY-876, which inhibit GLUT1/GLUT4, have the ability to target crucial transporters involved in nutrient uptake (Fig. 5) [123, 136]. Inhibiting these transporters can deprive cancer cells of essential nutrients, limiting their ability to adapt and survive in nutrient-deprived conditions. Furthermore, strategies aimed at disrupting the ability of cancer cells to utilize alternative fuel sources, such as glutamine and fatty acids, have also shown promise. For example, inhibiting enzymes involved in glutamine metabolism, such as BPTES, can reduce the availability of this nutrient and impair cancer cell growth. Similarly, UCM05 and TOFA have the potential to deprive cancer cells of an important source of energy by inhibiting fatty acid metabolizing enzymes (Fig. 5).

Compensatory metabolic reprogramming in cancer aims to enable the survival and growth of cancer cells by counteracting the effects of therapies that target their uncontrolled proliferation [146]. Some drugs have been developed based on this mechanism and the target, and encouragingly, some have been tested in clinical trials (Table 2) [147, 148].

**Table 2:** Clinical studies evaluating drugs that target cancer metabolism

Target	Drugs	Cancer	Clinical Trials	Status	ID
GLS1	CB-839	Advanced cervical cancer	Phase I	Active	NCT05521997
GLS1	CB-839	Specific genetic mutations and solid tumors or malignant peripheral nerve sheath tumors	Phase II	Active	NCT03872427
GLS1	CB-839	Prostate cancer	Phase II	Active	NCT04824937
GLS1	CB-839	Recurrent or refractory multiple myeloma	Phase I	Active	NCT03798678
GLS1	CB-839	IDH-mutated diffuse astrocytoma or anaplastic astrocytoma	Phase Ib	Active	NCT03528642
PDH/α-KGDH	CPI-613	Leukemia, lymphoma	phase II	Active	NCT03793140
PDH/α-KGDH	CPI-613	Pancreatic cancer	Phase I/II	Active	NCT03699319
PDH/α-KGDH	CPI-613	Biliary tract cancer	Phase I/II	Active	NCT04203160
OXPHOS	Metformin	Malignant glioma	Phase II	Active	NCT04945148
OXPHOS	ME-344	Previously treated metastatic colorectal cancer	Phase Ib	Active	NCT05824559
FASN	TVB-2640	Colon and other resectable cancers	Phase I	Active	NCT02980029
FASN	TVB-2640	Castration-resistant prostatic neoplasms,	Phase I	Active	NCT05743621
FASN	Omeprazole	Prostate cancer, refractory Cancer, castration resistant prostatic cancer	Phase II	Active	NCT04337580
LAT1	IAG933	Mesothelioma	Phase I	Active	NCT04857372
LAT1	JPH203	Advanced biliary tract cancers	Phase II	Active	JPRN-UMIN000034080

CB-839 is a glutaminase 1 (GLS1) inhibitor that has been investigated for cervical cancer (NCT05521997), prostate cancer (NCT04824937), and peripheral nerve sheath tumors (NCT03872427) in phase I or phase II trials. TVB-2640 inhibits FASN and has also entered a phase I clinical trial of colon and other resectable cancers (NCT02980029). Combinations with other chemotherapy or immune drugs can strengthen the therapeutic effect [149, 150]. ME-344 is another OXPHOS inhibitor which, combined with bevacizumab, has been used in metastatic colorectal cancer (NCT05824559). Considering the metabolic heterogeneity of cancer cells and the conversion of multiple metabolic pathways, inhibitory drugs combined with multiple metabolic pathways can achieve better results [151, 152]. For example, by inhibiting lipolysis and glucose metabolism, jointly targeting glycolysis and glutamine metabolism is a current research direction. In recent studies, it was discovered that the simultaneous implementation of glutamine starvation and the glycolysis inhibitor 2-deoxy-D-glucose resulted in a more pronounced cytotoxic effect (Fig. 5) [153].

## Conclusions

Cancer cell metabolism is a complex and dynamic process that contributes to the uncontrolled growth and survival of cancer cells. Different metabolic phenotypes exist in different types of cancer and are dependent on different metabolic patterns [154]. Dysregulation of cancer cell metabolism is driven by diverse genetic and epigenetic alterations that affect key metabolic genes and signaling networks. The guiding principle for choosing therapeutic agents for cancers with diverse metabolic characteristics is to target specific metabolic pathways that are dysregulated in different cell populations. Therefore, a strategy involving the simultaneous targeting of multiple metabolic patterns can be pursued by targeting various genes and pathways with a combination of drugs. This can be achieved by understanding the diverse metabolic profiles within the tumor and identifying the crucial pathways that drive tumor growth, survival and metastasis. Based on this, drugs can be identified that selectively block or interfere with these metabolic pathways, resulting in targeted elimination of cancer cells exhibiting metabolic dysregulation [155]. In addition, therapies that can exploit the metabolic vulnerabilities of cancer cells, including those involved in cellular energy production or nutrient absorption, should be considered. Ultimately, the goal is to develop personalized medicine approaches that take into account the metabolic heterogeneity of tumor tissues and target the specific metabolic abnormalities responsible for driving tumor development and progression.

## Data Availability

Not applicable.

## Abbreviations

ACC: Acetyl-CoA carboxylase
ACLY: ATP citrate lyase
ASCT2: Alanine serine cysteine transporter 2
BCAA: Branched-chain amino acids
CAFs: Cancer-associated fibroblasts
CPT1: Carnitine palmitoyltransferase 1
CSCs: Cancer stem cells
ECM: Extracellular matrix
EMT: Epithelial-mesenchymal transition
FAO: Fatty acid oxidation
FASN: Fatty acid synthase
FH: Fumarate hydratase
GLS1: Glutaminase 1
GSH: Glutathione
HBP: Hexosamine biosynthetic pathway
HK2: Hexokinase 2
LAT1: L-type amino acid transporter 1
IDH: Isocitrate dehydrogenase
MCT1: Monocarboxylate transporter 1
MMPs: Matrix metalloproteinases
mTOR: Mammalian target of the rapamycin
MUFAs: Monounsaturated fatty acids
NK: Natural killer
OXPHOS: Oxidative phosphorylation
PFK-2: 6-phosphofructo-2-kinase
PPP: Pentose phosphate pathway
ROS: Reactive oxygen species
SCD1: Stearoyl-CoA desaturase 1
SDH: Succinate dehydrogenase
SFAs: Saturated fatty acids
TAMs: Tumor-associated macrophages
TCA: Tricarboxylic acid
TME: Tumor microenvironment
UC: Urea cycle
VEGF: Vascular endothelial growth factor
